# A proposal for the reference intervals of the Italian microbiota “scaffold” in healthy adults

**DOI:** 10.1038/s41598-022-08000-x

**Published:** 2022-03-10

**Authors:** Davide Sisti, Valerio Pazienza, Fabio Piccini, Barbara Citterio, Wally Baffone, Sabrina Donati Zeppa, Francesca Biavasco, Emilia Prospero, Antonio De Luca, Marco Artico, Samanta Taurone, Andrea Minelli, Francesco Perri, Elena Binda, Riccardo Pracella, Riccardo Santolini, Stefano Amatori, Piero Sestili, Marco B. L. Rocchi, Pietro Gobbi

**Affiliations:** 1 Department of Biomolecular Sciences, University of Urbino Carlo Bo, Piazza Rinascimento 7, 61029 Urbino, Italy; 2 Division of Gastroenterology, IRCCS “Casa Sollievo della Sofferenza” Hospital, 71013 San Giovanni Rotondo, Italy; 3 Italian Microbiome Project, 35100 Padua, Italy; 4 Department of Life and Environmental Sciences, Università Politecnica delle Marche, 60121 Ancona, Italy; 5 Department of Biomedical Sciences, Università Politecnica delle Marche, 60121 Ancona, Italy; 6 Department of Mental and Physical Health and Preventive Medicine, Section of Human Anatomy, University of Campania “Luigi Vanvitelli”, 80138 Naples, Italy; 7 Department of Sense Organs, La Sapienza University, 00185 Rome, Italy; 8 Cancer Stem Cells Unit ISBReMIT, IRCCS “Casa Sollievo della Sofferenza” Hospital, 71013 San Giovanni Rotondo, Italy; 9 Department of Humanities, University of Urbino Carlo Bo, 61029 Urbino, Italy

**Keywords:** Bacteria, Microbial communities, Prokaryote

## Abstract

Numerous factors, ranging from genetics, age, lifestyle, and dietary habits to local environments, contribute to the heterogeneity of the microbiota in humans. Understanding the variability of a “healthy microbiota” is a major challenge in scientific research. The gut microbiota profiles of 148 healthy Italian volunteers were examined by 16S rRNA gene sequencing to determine the range and diversity of taxonomic compositions in the gut microbiota of healthy populations. Possible driving factors were evaluated through a detailed anamnestic questionnaire. Microbiota reference intervals were also calculated. A “scaffold” of a healthy Italian gut microbiota composition was identified. Differences in relative quantitative ratios of microbiota composition were detected in two clusters: a bigger cluster (C_2_), which included 124 subjects, was characterized by more people from the northern Italian regions, who habitually practised more physical activity and with fewer dietary restrictions. Species richness and diversity were significantly higher in this cluster (C_2_) than in the other one (C_1_) (C_1_: 146.67 ± 43.67; C_2_: 198.17 ± 48.47; *F* = 23.40; *P* < 0.001 and C_1_: 16.88 ± 8.66; C_2_: 35.01 ± 13.40; *F* = 40.50; *P* < 0.001, respectively). The main contribution of the present study was the identification of the existence of a primary healthy microbiological framework that is only marginally affected by variations. Taken together, our data help to contextualize studies on population-specific variations, including marginal aspects, in human microbiota composition. Such variations must be related to the primary framework of a healthy microbiota and providing this perspective could help scientists to better design experimental plans and develop strategies for precision tailored microbiota modulation.

## Introduction

The microbial communities colonizing different body districts comprise trillions of microorganisms that perform vital functions and play a role in keeping us healthy^1^. The human gut microbiota is composed of bacteria, archaea, fungi, protozoans, and viruses, particularly bacteriophages, probably due to the prevalence of bacteria in this environment^2^. The microbiota can be viewed as a community composed of autochthonous or resident microorganisms and allochthonous or transient microorganisms^3^. Bacteria, mostly anaerobic, are predominant in this environment and consist of two main phylotypes: *Bacteroidetes*, including the genera *Prevotella* and *Bacteroides*, and *Firmicutes*, including *Clostridium* clusters and members of *Eubacterium, Faecalibacterium*, *Roseburia*, and *Ruminococcus*. On the other hand, *Proteobacteria*, *Actinobacteria*, *Fusobacteria*, and *Verrucomicrobia* phyla are present in relatively small numbers^4^. Various roles are attributed to the microbial community, including immune system maintenance, vitamin production, digestion, energy homeostasis, angiogenesis, metabolite synthesis, and the maintenance of intestinal barrier integrity^5^. The gut microbiota develops in children between the ages of one and three and remains relatively stable throughout life. During the transition from childhood to adulthood, the genera *Bifidobacteria* decrease, while *Bacteroidetes* increase, affecting the gut's metabolic activity and health. Several studies have reported that *Bifidobacteria* are beneficial, playing a role in protecting the gut epithelium, while *Firmicutes*, particularly *Clostridia* and *Enterobacteriaceae*, whose numbers increase in elderly subjects, are considered detrimental^6^.

Although microbiota evolves throughout the lifetime, it is now recognized that one-third of the gut microbiota is common to most people, whereas the remaining two-thirds are specific to each individual. In particular, at lower taxonomic levels (i.e., species), the microbiota is influenced by several individual factors, such as type of delivery at birth and the method of infant feeding, the use or abuse of medications, especially antibiotics, diet, supplements, lifestyle habits (smoking, physical activity) etc^1,7,8^. Recent studies have highlighted emerging differences in microbiota composition even in population cohorts with similar genetic and cultural backgrounds^9–11^. The balance between the two most important phyla found in the gut, *Firmicutes* and *Bacteroidetes*, is essential to maintaining homeostasis in the host and, consequently, health.

Considering the central role played by intestinal microbiota in modulating several pathological disorders and response to treatments, investigating their population-specific variations may lead to findings that contribute to enhancing the benefits of existing diagnostic and therapeutic strategies^12–14^. Identifying and classifying specific sets of microbiota features that promote health is an essential first step to correcting microbial configurations implicated in disease. In fact, although attention is already being focused on how to manipulate microbiota to improve health status, there is a consensus within the scientific community that studying the factors constituting the normal ranges of these features in healthy populations is of fundamental importance^15^. Despite several important papers have been published on the microbiota composition of Italians suffering from specific diseases or as a changing ecosystem, few data are available on the microbiota of the healthy population^1,9,16^. Hence, our study aimed to define the reference intervals of the gut microbiota of a sample of Italian subjects with relatively homogeneous physiological features.

## Results

Samples of 148 participants (M: 69, F: 79; age: 39.8 ± 16.8 years; height: 164.4 ± 18.3 cm; weight: 61.2 ± 17.5 kg; body mass index: 22.0 ± 4.1 kg/m^2^) were collected and analysed. Of the 148 subjects, 22 were under 18 years old, 16 were smokers, 35 were ex-smokers, and 97 were non-smokers. In addition, 104 practised sports. Regarding diet, 93 participants had a typical Mediterranean diet, 15 were vegetarian, 4 were vegan, 4 were on a paleo diet, and the remaining 32 reported following another type of diet. Finally, 15 subjects reported avoiding eating certain foods for dietary or ethical reasons, and 27 had other kinds of sporadic food restrictions. The sample size was large enough for the aim of the paper. Indeed, the total number of genera found in the gut pan-microbiota increased with the number of samples, as shown in Supplementary Fig. S1.

### Subject clustering

**Figure 1:**
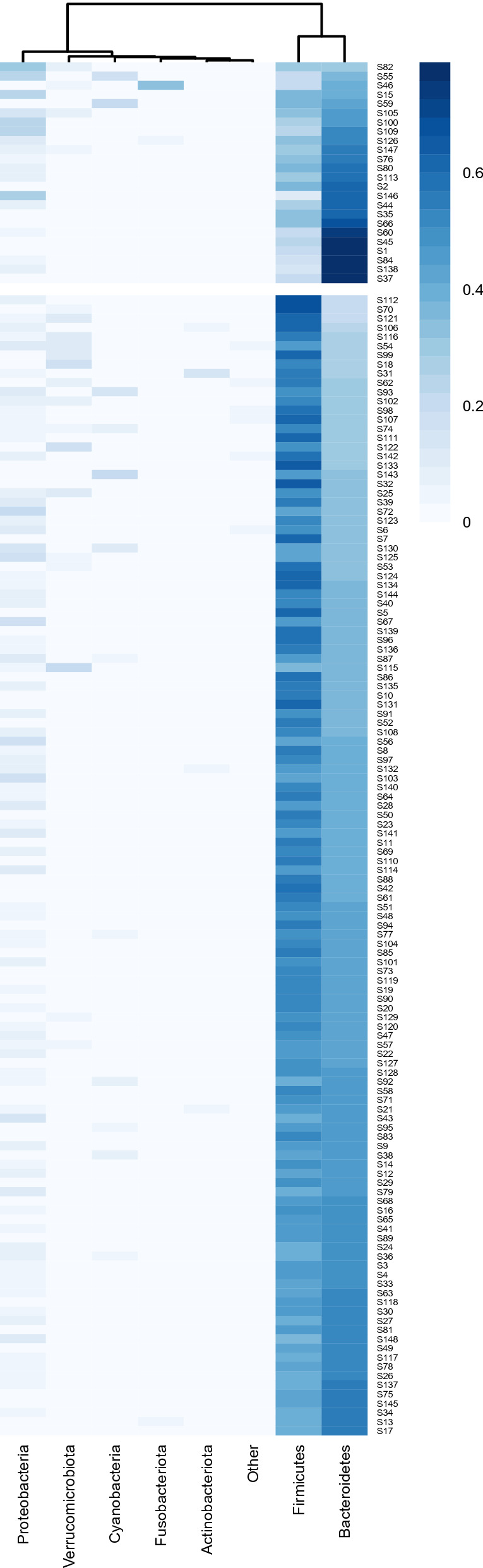
Heatmap of the most represented phyla. Each column represents a single phylum, with each row representing a different subject/sample. Bray–Curtis distance was used as a clustering method. Two different clusters of subjects were selected as the best solution (see above).

The analysis performed using the elbow and silhouette methods showed that the best clustering solution was the one with two clusters of subjects; both methods yielded the same results (Supplementary Fig. S2). The two clusters are clearly defined: Cluster 1 (C_1_) comprises 24 subjects, while Cluster 2 (C_2_) includes 124 subjects. Differences were observed between the two clusters in terms of phyla (*F* = 72.42), families (*F* = 9.43), and genera (*F* = 4.94) (PERMANOVA, Bray–Curtis dissimilarity index, Bonferroni corrected; *P* < 0.001 for all comparisons). Heatmap was used to represent the abundance of the most prevalent phyla. *Firmicutes* and *Bacteroidetes* together constituted more than 85% of the total phyla abundance. The difference between the two clusters, C_1_ (Fig. 1 above) and C_2_ (Fig. 1 below), is evident: *Bacteroidetes* were prevalent in C_1_, while *Firmicutes* were prevalent in C_2_.

### General features of the population

The subjects in the two clusters had similar characteristics, as was shown by the comparison reported in Table 1. No significant differences were found between the two clusters in terms of gender, body mass index, diet type, smoking habits, or alcohol consumption. Regarding food restrictions, no significant difference was found for lactose "intolerance" (subject's self–definition, in the absence of any clinical diagnosis) between the two clusters. By contrast, a three-fold increase was found for other food restrictions in the subjects in C_1_ (41.7% C_1_ vs 13.7% C_2_, *P* = 0.003). The macro-region of origin (north vs centre vs south) also showed a difference in the distribution between the two clusters, with C_2_ being comprising almost a doubled proportion of subjects living in northern Italy with respect to C_1_ (62.1% vs 33.3%, respectively), and a lower percentage of subjects living in central Italy (29.8% vs 54.2%, respectively) (*P* = 0.03). Finally, physical activity habits differed between the two clusters, with a higher proportion of subjects in C_2_ who declared practising more than 4 h/week of physical activity with respect to C_1_ (26.6% vs 8.3%, respectively; *P* = 0.04).

**Table 1:** The sociodemographic characteristics of the subjects in the two clusters.

	Cluster 1 (N = 24)	Cluster 2 (N = 124)	*p* (Cramer’s V)
**Gender**			
Male	12 (50.0%)	57 (46.0%)	0.72 (0.03)
Female	12 (50.0%)	67 (54.0%)	
**Age**			
< 18	4 (16.7%)	18 (14.5%)	0.68 (0.08)
18–65	20 (83.3%)	101 (81.5%)	
> 65	0 (0.0%)	5 (4.0%)	
**Body mass index (kg/m**^**2**^**)**			
< 18.5	4 (16.7%)	18 (14.5%)	1.00 (0.02)
18.5–25	15 (62.5%)	80 (64.5%)	
> 25	5 (20.8%)	26 (21.0%)	
**Physical activity**			
< 1 h/week	7 (29.2%)	45 (36.3%)	**0.04 (0.21)**
1–4 h/week	15 (62.5%)	46 (37.1%)	
> 4 h/week	2 (8.3%)	33 (26.6%)	
**Region of residence**			
North	8 (33.3%)	77 (62.1%)	**0.03 (0.22)**
Central	13 (54.2%)	37 (29.8%)	
South	3 (12.5%)	10 (8.1%)	
**Type of residence**			
City	15 (62.5%)	90 (72.6%)	0.33 (0.08)
Countryside	9 (37.5%)	34 (27.4%)	
**Smoker**			
Yes	4 (16.7%)	12 (9.7%)	0.38 (0.11)
Ex-smoker	7 (29.2%)	28 (22.6%)	
No	13 (54.2%)	84 (67.7%)	
**Food restrictions**			
Lactose restriction			
Yes	4 (16.7%)	11 (8.9%)	0.27 (0.10)
No	20 (83.3%)	113 (91.1%)	
Other food restrictions			
Yes	10 (41.7%)	17 (13.7%)	**0.003 (0.27)**
No	14 (58.3%)	107 (86.3%)	
**Diet**			
Mediterranean	12 (50.0%)	81 (65.3%)	0.29 (0.13)
Vegetarian/Vegan	5 (20.8%)	14 (11.3%)	
Other	7 (29.2%)	29 (23.4%)	
**Breast feeding**			
Yes	21 (87.5%)	102 (82.3%)	0.58 (0.05)
No	3 (12.5%)	22 (17.7%)	
**Animals**			
Yes	14 (58.3%)	55 (44.4%)	0.21 (0.10)
No	10 (41.7%)	69 (55.6%)	
**Alcohol (last 2 days)**			
Yes	9 (37.5%)	43 (34.7%)	0.79 (0.02)
No	15 (62.5%)	81 (65.3%)	

Significant values are in bold.

### The microbial diversity and richness of the two clusters

In addition to the presence of food restrictions in C_2_, the cluster is also characterized by higher species richness (OTU number: C_1_ = 146.67 ± 43.67; C_2_ = 198.17 ± 48.47; *F*_(1,146)_ = 23.40; *P* < 0.001) and diversity (Shannon effective number of species: C_1_ = 16.88 ± 8.66; C_2_ = 35.01 ± 13.40; *F*_(1,146)_ = 40.50; *P* < 0.001) when compared to C_1_.

### Microbial community composition

**Figure 2:**
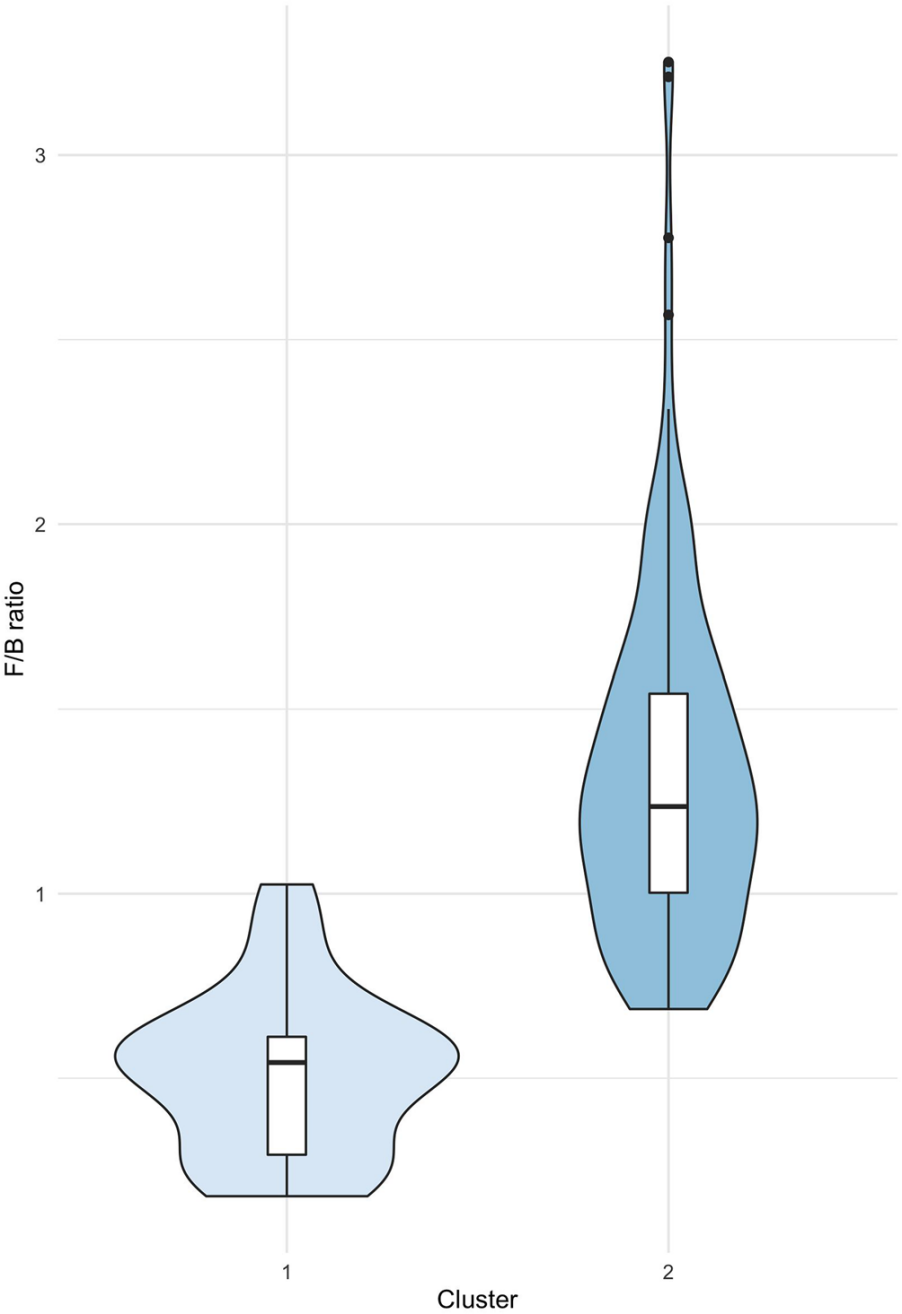
Violin plots of the *Firmicutes/Bacteroidetes* ratio in the two clusters.

The microbiota profile of 148 Italian volunteers were characterized at the taxonomic level (*i.e.,* phylum, family, and genus; Table 2). The taxonomic assignment of the V3-V4 hypervariable region of the 16S rRNA gene showed *Firmicutes* (46.5%) and *Bacteroidetes* (43.2%) to be the predominant phyla. *Proteobacteria* (6.2%) were less abundant, while the remaining phyla were rarely detected, constituting altogether 4.2% of the total population. The analysis of the two clusters shows a higher percentage of *Bacteroidetes* (57.5% vs 40.4%) and a lower percentage of *Firmicutes* (26.9% vs 50.2%) in C_1_ compared to C_2_; hence, there was a higher *Firmicutes/Bacteroidetes* ratio in C_2_ than in C_1_, as shown in Fig. 2 (*F/B* Ratio: C_1_ 0.51 ± 0.22, C_2_ 1.33 ± 0.48; Mann–Whitney *U* = 73.0; *P* < 0.001).

**Table 2:** Mean, upper and lower limits of 95% reference intervals are reported for phyla, families, and genera with abundance > 0.1%.

	Total	Cluster 1	Cluster 2
**Phyla**			
*P: Firmicutes*	46.45% (18.05–66.11)	26.99% (3.75–37.38)	50.21% (36.53–66.01)
*P: Bacteroidetes*	43.17% (20.94–89.29)	57.55% (21.76–80.83)	40.38% (17.8–56.85)
*P: Proteobacteria*	6.15% (0.56–30.17)		
*P: Verrucomicrobiota*	1.6% (0–17.41)		
*P: Cyanobacteria*	1.16% (0–21.28)		
*P: Actinobacteriota*	0.6% (0–4.74)	0.3% (0–3.16)	0.66% (0–4.83)
*P: Fusobacteriota*	0.29% (0–2.45)		
*P: Euryarchaeota*	0.24% (0–4.27)		
*P: Desulfobacterota*	0.17% (0–1.59)		
**Families**			
*P: Bacteroidetes; C: Bacteroidia; O: Bacteroidales; F: Bacteroidaceae*	25.93% (1.09–67.09)		
*P: Firmicutes; C: Clostridia; O: Lachnospirales; F: Lachnospiraceae*	21.8% (3.22–41.47)	13.55% (3.32–23.15)	23.39% (4.42–41.24)
*P: Bacteroidetes; C: Bacteroidia; O: Bacteroidales; F: Prevotellaceae*	10.45% (0–73.71)		
*P: Firmicutes; C: Clostridia; O: Oscillospirales; F: Ruminococcaceae*	9.96% (0.56–27.93)	6.08% (0–13.42)	10.71% (0.32–28.56)
*P: Firmicutes; C: Clostridia; O: Oscillospirales; F: Oscillospiraceae*	2.53% (0.01–15.98)	0.98% (0–5.43)	2.83% (0.12–15.49)
*P: Proteobacteria; C: Gammaproteobacteria; O: Burkholderiales; F: Sutterellaceae*	2.26% (0–10.88)		
*P: Bacteroidetes; C: Bacteroidia; O: Bacteroidales; F: Tannerellaceae*	2.21% (0–12.91)		
*P: Proteobacteria; C: Alphaproteobacteria; O: Rhodospirillales; F: uncultured*	2.18% (0–17.76)		
*P: Bacteroidetes; C: Bacteroidia; O: Bacteroidales; F: Rikenellaceae*	2.16% (0–9.91)		
*P: Firmicutes; C: Clostridia; O: Clostridia_UCG-014; F: Clostridia_UCG-014*	1.94% (0–13.12)	0.64% (0–4.63)	2.19% (0–13.12)
*P: Verrucomicrobiota; C: Verrucomicrobiae; O: Verrucomicrobiales; F: Akkermansiaceae*	1.51% (0–17.41)		
*P: Firmicutes; C: Negativicutes; O: Acidaminococcales; F: Acidaminococcaceae*	1.28% (0–8.02)		
*P: Proteobacteria; C: Gammaproteobacteria; O: Enterobacterales; F: Enterobacteriaceae*	1.24% (0–31.06)		
*P: Cyanobacteria; C: Vampirivibrionia; O: Gastranaerophilales; F: Gastranaerophilales*	1.15% (0–21.28)		
*P: Firmicutes; C: Clostridia; O: Clostridia_vadinBB60_group; F: Clostridia_vadinBB60_group*	1.15% (0–8.73)	0.24% (0–1.85)	1.32% (0–9.76)
*P: Firmicutes; C: Clostridia; O: Christensenellales; F: Christensenellaceae*	1.1% (0–7.76)	0.3% (0–6.38)	1.25% (0–8.44)
*P: Bacteroidetes; C: Bacteroidia; O: Bacteroidales; F: Barnesiellaceae*	1.03% (0–5.14)		
*P: Firmicutes; C: Clostridia; O: Oscillospirales; F: [Eubacterium]_coprostanoligenes_group*	0.88% (0–5.58)	0.39% (0–2.72)	0.97% (0–6.59)
*P: Firmicutes; C: Negativicutes; O: Veillonellales-Selenomonadales; F: Veillonellaceae*	0.87% (0–6.53)		
*P: Bacteroidetes; C: Bacteroidia; O: Bacteroidales; F: Marinifilaceae*	0.75% (0–2.38)		
*P: Firmicutes; C: Bacilli; O: Erysipelotrichales; F: Erysipelatoclostridiaceae*	0.74% (0.02–5.48)	0.28% (0–1.88)	0.83% (0.01–8.81)
*P: Firmicutes; C: Clostridia; O: Monoglobales; F: Monoglobaceae*	0.7% (0–3.66)		
*P: Firmicutes; C: Clostridia; O: Oscillospirales; F: UCG-010*	0.68% (0–5.74)	0.3% (0–9.27)	0.76% (0–6.48)
*P: Bacteroidetes; C: Bacteroidia; O: Bacteroidales; F: Muribaculaceae*	0.56% (0–6.46)		
*P: Firmicutes; C: Clostridia; O: Oscillospirales; F: Butyricicoccaceae*	0.48% (0.02–2.08)	0.24% (0–1.07)	0.53% (0.01–2.04)
*P: Actinobacteriota; C: Actinobacteria; O: Bifidobacteriales; F: Bifidobacteriaceae*	0.48% (0–3.67)	0.22% (0–2.71)	0.53% (0–4.24)
*P: Firmicutes; C: Bacilli; O: Erysipelotrichales; F: Erysipelotrichaceae*	0.36% (0–3.93)		
*P: Firmicutes; C: Bacilli; O: Izemoplasmatales; F: Izemoplasmatales*	0.35% (0–4.88)	0.02% (0–0.44)	0.41% (0–6.95)
*P: Firmicutes; C: Clostridia; O: Peptostreptococcales-Tissierellales; F: Peptostreptococcaceae*	0.3% (0–1.96)		
*P: Fusobacteriota; C: Fusobacteriia; O: Fusobacteriales; F: Fusobacteriaceae*	0.29% (0–2.45)		
*P: Proteobacteria; C: Gammaproteobacteria; O: Pasteurellales; F: Pasteurellaceae*	0.26% (0–2.86)		
*P: Euryarchaeota; C: Methanobacteria; O: Methanobacteriales; F: Methanobacteriaceae*	0.24% (0–4.19)		
*P: Firmicutes; C: Clostridia; O: Clostridiales; F: Clostridiaceae*	0.23% (0–1.49)		
*P: Firmicutes; C: Bacilli; O: Acholeplasmatales; F: Acholeplasmataceae*	0.23% (0–4.75)		
*P: Firmicutes; C: Bacilli; O: Lactobacillales; F: Streptococcaceae*	0.19% (0–1.65)		
*P: Firmicutes; C: Bacilli; O: RF39; F: RF39*	0.18% (0–2.99)	0.02% (0–0.41)	0.21% (0–3.06)
*P: Desulfobacterota; C: Desulfovibrionia; O: Desulfovibrionales; F: Desulfovibrionaceae*	0.17% (0–1.57)		
*P: Proteobacteria; C: Gammaproteobacteria; O: Aeromonadales; F: Succinivibrionaceae*	0.15% (0–0.05)		
*P: Firmicutes; C: Clostridia; O: Peptostreptococcales-Tissierellales; F: Anaerovoracaceae*	0.14% (0–0.93)		
**Genera**			
*P: Bacteroidetes; C: Bacteroidia; O: Bacteroidales; F: Bacteroidaceae; G: Bacteroides*	25.93% (1.09–67.09)		
*P: Bacteroidetes; C: Bacteroidia; O: Bacteroidales; F: Prevotellaceae; G: Prevotella*	7.98% (0–70.33)		
*P: Firmicutes; C: Clostridia; O: Oscillospirales; F: Ruminococcaceae; G: Faecalibacterium*	4.07% (0.34–20.59)		
*P: Firmicutes; C: Clostridia; O: Lachnospirales; F: Lachnospiraceae; G: Unclassified*	2.61% (0.08–7.66)	1.51% (0–4.36)	2.82% (0.31–7.83)
*P: Firmicutes; C: Clostridia; O: Lachnospirales; F: Lachnospiraceae; G: Roseburia*	2.58% (0–12.04)		
*P: Firmicutes; C: Clostridia; O: Lachnospirales; F: Lachnospiraceae; G: Lachnospira*	2.57% (0–15.38)		
*P: Bacteroidetes; C: Bacteroidia; O: Bacteroidales; F: Tannerellaceae; G: Parabacteroides*	2.21% (0–12.78)		
*P: Proteobacteria; C: Alphaproteobacteria; O: Rhodospirillales; F: uncultured; G: uncultured*	2.18% (0–17.76)		
*P: Firmicutes; C: Clostridia; O: Lachnospirales; F: Lachnospiraceae; G: Blautia*	2.02% (0.31–7.38)		
*P: Bacteroidetes; C: Bacteroidia; O: Bacteroidales; F: Rikenellaceae; G: Alistipes*	1.97% (0–9.89)		
*P: Firmicutes; C: Clostridia; O: Clostridia_UCG-014; F: Clostridia_UCG-014; G: Clostridia_UCG-014*	1.94% (0–13.28)	0.64% (0–4.63)	2.19% (0–13.12)
*P: Firmicutes; C: Clostridia; O: Oscillospirales; F: Ruminococcaceae; G: Ruminococcus*	1.9% (0–12.56)	1.1% (0–12.59)	2.05% (0–16.77)
*P: Firmicutes; C: Clostridia; O: Lachnospirales; F: Lachnospiraceae; G: Lachnospiraceae_NK4A136_group*	1.83% (0–11.57)	0.97% (0–7.16)	2% (0–11.99)
*P: Verrucomicrobiota; C: Verrucomicrobiae; O: Verrucomicrobiales; F: Akkermansiaceae; G: Akkermansia*	1.51% (0–17.41)		
*P: Firmicutes; C: Clostridia; O: Lachnospirales; F: Lachnospiraceae; G: [Eubacterium]_eligens_group*	1.42% (0–8.07)	0.75% (0–4.08)	1.55% (0–8.3)
*P: Proteobacteria; C: Gammaproteobacteria; O: Burkholderiales; F: Sutterellaceae; G: Sutterella*	1.36% (0–6.15)		
*P: Firmicutes; C: Clostridia; O: Lachnospirales; F: Lachnospiraceae; G: Agathobacter*	1.16% (0–6.4)		
*P: Firmicutes; C: Clostridia; O: Lachnospirales; F: Lachnospiraceae; G: Coprococcus*	1.15% (0–7.53)	0.47% (0–2.96)	1.28% (0–7.98)
*P: Cyanobacteria; C: Vampirivibrionia; O: Gastranaerophilales; F: Gastranaerophilales; G: Gastranaerophilales*	1.15% (0–21.28)		
*P: Firmicutes; C: Negativicutes; O: Acidaminococcales; F: Acidaminococcaceae; G: Phascolarctobacterium*	1.15% (0–8.55)		
*P: Firmicutes; C: Clostridia; O: Clostridia_vadinBB60_group; F: Clostridia_vadinBB60_group; G: Clostridia_vadinBB60_group*	1.15% (0–8.73)	0.24% (0–1.85)	1.32% (0–9.76)
*P: Firmicutes; C: Clostridia; O: Oscillospirales; F: Ruminococcaceae; G: [Eubacterium]_siraeum_group*	1.13% (0–9.32)		
*P: Proteobacteria; C: Gammaproteobacteria; O: Enterobacterales; F: Enterobacteriaceae; G: Escherichia-Shigella*	1.13% (0–28.31)		
*P: Firmicutes; C: Clostridia; O: Christensenellales; F: Christensenellaceae; G: Christensenellaceae_R-7_group*	1.08% (0–7.63)	0.29% (0–6.35)	1.23% (0–8.17)
*P: Bacteroidetes; C: Bacteroidia; O: Bacteroidales; F: Prevotellaceae; G: Prevotellaceae_NK3B31_group*	1.07% (0–18.99)		
*P: Firmicutes; C: Clostridia; O: Oscillospirales; F: Oscillospiraceae; G: UCG-002*	1.04% (0–9.18)	0.3% (0–2.77)	1.18% (0–11.44)
*P: Proteobacteria; C: Gammaproteobacteria; O: Burkholderiales; F: Sutterellaceae; G: Parasutterella*	0.91% (0–12.06)		
*P: Firmicutes; C: Clostridia; O: Oscillospirales; F: [Eubacterium]_coprostanoligenes_group; G: [Eubacterium]_coprostanoligenes_group*	0.88% (0–5.58)	0.39% (0–2.72)	0.97% (0–6.59)
*P: Firmicutes; C: Clostridia; O: Oscillospirales; F: Ruminococcaceae; G: Subdoligranulum*	0.84% (0–4.84)	0.33% (0–3.53)	0.94% (0–4.88)
*P: Bacteroidetes; C: Bacteroidia; O: Bacteroidales; F: Barnesiellaceae; G: Barnesiella*	0.79% (0–3.99)		
*P: Firmicutes; C: Clostridia; O: Lachnospirales; F: Lachnospiraceae; G: Fusicatenibacter*	0.73% (0–4.78)	0.31% (0–3.19)	0.82% (0.01–5.18)
*P: Firmicutes; C: Negativicutes; O: Veillonellales-Selenomonadales; F: Veillonellaceae; G: Dialister*	0.7% (0–6.14)		
*P: Firmicutes; C: Clostridia; O: Monoglobales; F: Monoglobaceae; G: Monoglobus*	0.7% (0–3.65)		
*P: Firmicutes; C: Clostridia; O: Oscillospirales; F: UCG-010; G: UCG-010*	0.68% (0–5.74)	0.3% (0–9.27)	0.76% (0–6.5)
*P: Firmicutes; C: Clostridia; O: Oscillospirales; F: Ruminococcaceae; G: Unclassified*	0.68% (0–5.09)		
*P: Firmicutes; C: Clostridia; O: Oscillospirales; F: Ruminococcaceae; G: CAG-352*	0.65% (0–7.56)		
*P: Bacteroidetes; C: Bacteroidia; O: Bacteroidales; F: Prevotellaceae; G: Alloprevotella*	0.62% (0–16.06)		
*P: Firmicutes; C: Clostridia; O: Lachnospirales; F: Lachnospiraceae; G: [Eubacterium]_ruminantium_group*	0.59% (0–8.19)		
*P: Firmicutes; C: Clostridia; O: Lachnospirales; F: Lachnospiraceae; G: Anaerostipes*	0.55% (0–3.16)	0.26% (0–0.69)	0.61% (0.01–3.43)
*P: Bacteroidetes; C: Bacteroidia; O: Bacteroidales; F: Muribaculaceae; G: Muribaculaceae*	0.55% (0–6.47)		
*P: Firmicutes; C: Clostridia; O: Lachnospirales; F: Lachnospiraceae; G: Dorea*	0.54% (0–1.88)		
*P: Actinobacteriota; C: Actinobacteria; O: Bifidobacteriales; F: Bifidobacteriaceae; G: Bifidobacterium*	0.48% (0–3.64)	0.22% (0–2.71)	0.53% (0–4.24)
*P: Firmicutes; C: Clostridia; O: Lachnospirales; F: Lachnospiraceae; G: Lachnoclostridium*	0.46% (0–2.32)		
*P: Bacteroidetes; C: Bacteroidia; O: Bacteroidales; F: Marinifilaceae; G: Odoribacter*	0.46% (0–1.76)		
*P: Bacteroidetes; C: Bacteroidia; O: Bacteroidales; F: Prevotellaceae; G: Paraprevotella*	0.46% (0–3.83)		
*P: Firmicutes; C: Clostridia; O: Oscillospirales; F: Oscillospiraceae; G: UCG-005*	0.45% (0–2.71)	0.17% (0–3.99)	0.5% (0–2.64)
*P: Firmicutes; C: Clostridia; O: Oscillospirales; F: Butyricicoccaceae; G: Butyricicoccus*	0.43% (0–2.16)	0.24% (0–1.07)	0.47% (0–2.19)
*P: Firmicutes; C: Clostridia; O: Oscillospirales; F: Ruminococcaceae; G: uncultured*	0.39% (0–2.45)		
*P: Firmicutes; C: Clostridia; O: Lachnospirales; F: Lachnospiraceae; G: Lachnospiraceae_UCG-001*	0.39% (0–3.69)	0.14% (0–1.19)	0.44% (0–3.78)
*P: Firmicutes; C: Bacilli; O: Erysipelotrichales; F: Erysipelatoclostridiaceae; G: Erysipelotrichaceae_UCG-003*	0.38% (0–2.04)	0.18% (0–1.96)	0.42% (0–2)
*P: Firmicutes; C: Clostridia; O: Lachnospirales; F: Lachnospiraceae; G: Lachnospiraceae_UCG-003*	0.37% (0–10.84)		
*P: Firmicutes; C: Bacilli; O: Izemoplasmatales; F: Izemoplasmatales; G: Izemoplasmatales*	0.35% (0–4.86)	0.02% (0–0.44)	0.41% (0–6.95)
*P: Firmicutes; C: Clostridia; O: Lachnospirales; F: Lachnospiraceae; G: [Eubacterium]_xylanophilum_group*	0.35% (0–1.58)	0.1% (0–1.35)	0.4% (0–1.62)
*P: Firmicutes; C: Clostridia; O: Oscillospirales; F: Oscillospiraceae; G: UCG-003*	0.33% (0–1.4)	0.14% (0–1.49)	0.36% (0–1.52)
*P: Firmicutes; C: Clostridia; O: Lachnospirales; F: Lachnospiraceae; G: Butyrivibrio*	0.32% (0–15.4)		
*P: Firmicutes; C: Clostridia; O: Lachnospirales; F: Lachnospiraceae; G: [Eubacterium]_hallii_group*	0.3% (0–0.97)	0.16% (0–0.86)	0.32% (0.01–1.1)
*P: Fusobacteriota; C: Fusobacteriia; O: Fusobacteriales; F: Fusobacteriaceae; G: Fusobacterium*	0.29% (0–2.45)		
*P: Bacteroidetes; C: Bacteroidia; O: Bacteroidales; F: Marinifilaceae; G: Butyricimonas*	0.28% (0–1.17)		
*P: Firmicutes; C: Clostridia; O: Oscillospirales; F: Oscillospiraceae; G: NK4A214_group*	0.27% (0–3)	0.04% (0–0.32)	0.31% (0–4.19)
*P: Firmicutes; C: Clostridia; O: Lachnospirales; F: Lachnospiraceae; G: Tyzzerella*	0.27% (0–4.34)		
*P: Firmicutes; C: Clostridia; O: Lachnospirales; F: Lachnospiraceae; G: [Ruminococcus]_torques_group*	0.26% (0.01–2)		
*P: Proteobacteria; C: Gammaproteobacteria; O: Pasteurellales; F: Pasteurellaceae; G: Haemophilus*	0.25% (0–2.78)		
*P: Firmicutes; C: Clostridia; O: Lachnospirales; F: Lachnospiraceae; G: [Eubacterium]_ventriosum_group*	0.24% (0–1.32)	0.1% (0–1.35)	0.27% (0–1.34)
*P: Firmicutes; C: Bacilli; O: Acholeplasmatales; F: Acholeplasmataceae; G: Anaeroplasma*	0.23% (0–4.75)		
*P: Firmicutes; C: Clostridia; O: Clostridiales; F: Clostridiaceae; G: Clostridium_sensu_stricto_1*	0.23% (0–1.5)		
*P: Firmicutes; C: Bacilli; O: Erysipelotrichales; F: Erysipelotrichaceae; G: Holdemanella*	0.23% (0–2.68)		
*P: Euryarchaeota; C: Methanobacteria; O: Methanobacteriales; F: Methanobacteriaceae; G: Methanobrevibacter*	0.22% (0–4.14)		
*P: Firmicutes; C: Clostridia; O: Lachnospirales; F: Lachnospiraceae; G: Lachnospiraceae_UCG-004*	0.19% (0–1.05)		
*P: Firmicutes; C: Bacilli; O: Erysipelotrichales; F: Erysipelatoclostridiaceae; G: Asteroleplasma*	0.19% (0–5.16)		
*P: Firmicutes; C: Bacilli; O: Lactobacillales; F: Streptococcaceae; G: Streptococcus*	0.18% (0–1.66)		
*P: Bacteroidetes; C: Bacteroidia; O: Bacteroidales; F: Rikenellaceae; G: Rikenellaceae_RC9_gut_group*	0.18% (0–2.95)		
*P: Firmicutes; C: Bacilli; O: RF39; F: RF39; G: RF39*	0.18% (0–2.92)	0.02% (0–0.41)	0.21% (0–3.06)
*P: Firmicutes; C: Clostridia; O: Lachnospirales; F: Lachnospiraceae; G: Lachnospiraceae_ND3007_group*	0.17% (0–0.79)	0.06% (0–0.37)	0.19% (0–0.94)
*P: Firmicutes; C: Clostridia; O: Lachnospirales; F: Lachnospiraceae; G: Lachnospiraceae_UCG-010*	0.17% (0–0.87)	0.08% (0–0.98)	0.18% (0–1.02)
*P: Firmicutes; C: Clostridia; O: Oscillospirales; F: Ruminococcaceae; G: Incertae_Sedis*	0.15% (0–1.14)	0.08% (0–0.51)	0.17% (0–1.26)
*P: Bacteroidetes; C: Bacteroidia; O: Bacteroidales; F: Prevotellaceae; G: uncultured*	0.15% (0–3.18)		
*P: Firmicutes; C: Clostridia; O: Lachnospirales; F: Lachnospiraceae; G: CAG-56*	0.15% (0–1.07)		
*P: Firmicutes; C: Clostridia; O: Peptostreptococcales-Tissierellales; F: Peptostreptococcaceae; G: Romboutsia*	0.15% (0–1.1)	0.07% (0–0.49)	0.16% (0–1.42)
*P: Bacteroidetes; C: Bacteroidia; O: Bacteroidales; F: Prevotellaceae; G: Prevotellaceae_UCG-001*	0.14% (0–3.37)		
*P: Proteobacteria; C: Gammaproteobacteria; O: Aeromonadales; F: Succinivibrionaceae; G: Succinivibrio*	0.14% (0–0)		
*P: Bacteroidetes; C: Bacteroidia; O: Bacteroidales; F: Barnesiellaceae; G: uncultured*	0.12% (0–1.67)		
*P: Firmicutes; C: Negativicutes; O: Acidaminococcales; F: Acidaminococcaceae; G: Acidaminococcus*	0.12% (0–2.86)		
*P: Firmicutes; C: Clostridia; O: Oscillospirales; F: Oscillospiraceae; G: Colidextribacter*	0.12% (0–0.59)		
*P: Bacteroidetes; C: Bacteroidia; O: Bacteroidales; F: Barnesiellaceae; G: Coprobacter*	0.11% (0–0.92)		
*P: Firmicutes; C: Clostridia; O: Oscillospirales; F: Oscillospiraceae; G: Oscillibacter*	0.11% (0–0.58)		
*P: Firmicutes; C: Clostridia; O: Peptostreptococcales-Tissierellales; F: Peptostreptococcaceae; G: Intestinibacter*	0.11% (0–1.03)		
*P: Firmicutes; C: Clostridia; O: Lachnospirales; F: Lachnospiraceae; G: [Ruminococcus]_gauvreauii_group*	0.1% (0–0.66)		

The reference intervals are reported for C_1_ and C_2_ if the abundance between the two clusters was significantly different (Mann–Whitney test with false discovery rate).

*P* phylum, *C* class, *O* order, *F* family, *G* genus.

At the family level, *Bacteroidaceae* (25.9%), *Lachnospiraceae* (21.8%), *Prevotellaceae* (10.5%), *Ruminococcaceae* (9.9%), and *Oscillospiraceae* (2.5%) were the most representative among the over two-hundred families that were detected. Among these, significant differences were detected between the two clusters for *Lachnospiraceae*, *Ruminococcaceae,* and *Oscillospiraceae*, all the three being more abundant in C_2_ with respect to C_1_. Among the genera, *Bacteroides* (25.9%), *Prevotella* (8.0%), and *Faecalibacterium* (4.1%) showed the highest percentages of abundance.

**Figure 3:**
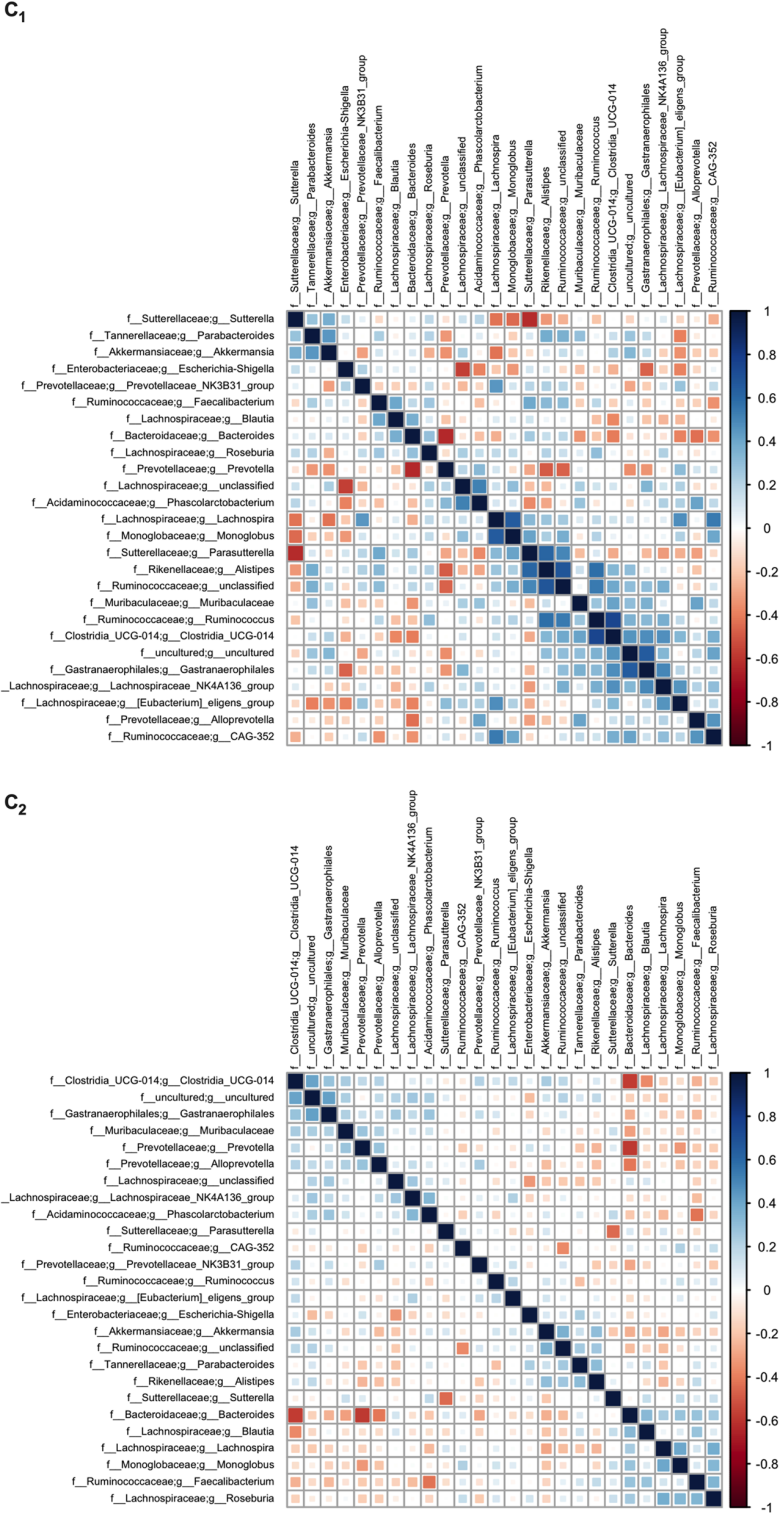
Spearman correlation plots of genera (cut off: relative abundance 0.5%) in the two clusters.

In Fig. 3, non-parametric correlation matrices are reported for all genera that showed an abundance greater than 0.5%, both for C_1_ (Fig. 3, upper plot) and C_2_ (Fig. 3, lower plot). In C_1_, some genera showed a strong negative correlation, as for *Prevotella* with *Bacteroides,* as well as *Sutterella* with *Lachnospira*, *Monoglobus*, and *Parasutterella*. On the contrary, some genera were found to be positively co-graduated. Overall, correlations detected in C_1_ should be interpreted with caution, as this cluster only comprises 24 subjects. As can be noted, C_2_ showed weaker correlations among genera with respect to C_1_, except for strongly negative values between genus *Bacteroides* and genus *Clostridia UCG-014*, and again, between *Bacteroides* and *Prevotella*.

## Discussion

Defining a healthy microbiota has become a major challenge for scientists. Although variations in the microbial community associated with a wide range of pathologies, from gastrointestinal disorders, autoimmune diseases, and cancer to mood disorders, have been documented, the composition and functional characteristics of a healthy microbiota have yet to be fully elucidated^12,13,17–20^. Defining the composition and functional characteristics of a healthy microbiota is so challenging because the microbiota is strongly influenced by a wide range of factors, including genetics; the mode of delivery at birth and the method of infant feeding; the use or abuse of medications and supplements, especially antibiotics, diet, lifestyle habits (smoking, physical activity), etc^1,7,8^. Recent studies have highlighted differences in microbiota composition even in population cohorts with similar genetic and cultural backgrounds^9–11^.

This study aimed to characterize the gut microbiota composition of healthy volunteers from Italy. The analysis of faecal samples provided information on the microbial composition that was in line with previously reported results^21^. The phyla *Firmicutes* and *Bacteroidetes* are indeed closely related to a healthy profile as well as a low number of species belonging to *Proteobacteria* phyla due to the anaerobic conditions in the colon^22^. Nevertheless, by k-means clustering, we were able to identify two main groups, C_1_ and C_2_, with the latter characterized by higher richness, diversity, and *Firmicutes/Bacteroidetes* ratio.

It is well known that most bacterial genera of the human gut microbiota belong to the phyla *Firmicutes* or *Bacteroidetes*, which account for about 90% of intestinal resident microorganisms^23^. *Firmicutes,* sub-grouped in *Clostridium coccoides* (*Clostridium* cluster XIVa) and *Clostridium leptum* (*Clostridium* cluster IV), are responsible for assimilating carbohydrates and animal fat, which are associated with the onset of obesity^24,25^. Among *Bacteroidetes,* the two prevalent genera in the human colon are *Bacteroides* and *Prevotella;* the former is highly associated with the consumption of animal proteins, amino acids, and saturated fats, which are typical components of the Western diet, and the latter with the consumption of complex carbohydrates and simple sugars, which are important components of vegetarian diets^26,27^.

Although several studies have attempted to define the composition of a healthy microbiota, such a definition remains elusive due to the many intrinsic and extrinsic factors influencing the gut ecosystem^28^. Nishijima et al.^29^ compared the compositions of the gut microbiomes of people from twelve different countries worldwide, showing great variations in the microbiome structure and function in healthy adults from different countries. At the genus level, the relative abundance of *Bacteroides* reported herein for the Italian population was similar to that reported for the United States, Canada, and Spain; similarly, the relative abundance of *Prevotella* was analogous to that which was reported for Canada, Denmark, Spain, and Russia. It can be noted that the microbiota composition found in this study in the healthy Italian population is similar to the composition reported in other countries with a predominantly Caucasian ethnicity^29^. The additional value of our study compared to similar investigations consists of quantifying reference intervals, which could have a direct application in diagnostics. Moreover, De Filippo et al. reported a significant enrichment in *Bacteroidetes* and a depletion of *Firmicutes* in African children whose diet was based on cereals, legumes, and vegetables and rich in carbohydrates, fibre, and non-animal protein^30^. The bacteria belonging to the genus *Bacteroides* are known to produce short-chain fatty acids (SCFA) and may thus contribute to preventing gut inflammation. Accordingly, multiple studies have reported an association between inflammatory bowel disease (IBD) and the flora disequilibrium of *Bacteroides*^31–34^.

In our study, *Firmicutes* showed a lower abundance than *Bacteroidetes* in C_1_. The greater relative abundance of *Bacteroidetes* suggests that in the intestines of those subjects, there may be a lower number of bacterial species favouring the onset of metabolic diseases such as those belonging to *Firmicutes*. In this regard, we also observed that the participants in this study, grouped according to their food habits and, to a lesser degree, according to their geographical origins, are mostly included in C_2_. Indeed, a significantly higher proportion of participants in C_1_ reported having food restrictions, mainly related to the consumption of dried and fresh vegetables. A limited consumption, or even worst, the exclusion of fresh vegetables and their derivatives from the diet, can induce alteration in the gut microbiota composition, leading to a reduction of fibre-degrading bacteria, able to produce greater amounts of SCFA^35,36^. Further, the heterogeneity of vegetables consumed has been positively correlated to the microbial alpha-diversity^37^. In addition, a significant difference in the distribution of the subjects among the Italian macro-regions (north, centre, south) was detected, with participants from the northern regions being more represented in the C_2_ and participants from the central regions being prevalent in the C_1_. This result is in accordance with those previously reported by Fontana and colleagues^9^, who detected differences in the gut microbiota composition of people pertaining to three different regions of Italy (one for each macro-area). Further, regular physical activity seems to be a discerning factor between the two clusters, with people who practice a higher volume of physical exercise (i.e., more than 4 h per week) being more present in the C_2_. This result is also coherent with a higher *Firmicutes/Bacteroidetes* ratio in this cluster, as this ratio was previously associated with higher cardiorespiratory fitness and athletic status^38^. This result is in accordance with other studies in the literature: Clarke et al.^39^ found a higher *Firmicutes/Bacteroidetes* ratio in a sample of rugby players compared to overweight controls; Huang et al.^40^ reported an increase in this ratio after six weeks of exercise and dietary restriction in obese adolescents; Donati Zeppa et al.^41^ found an increase in the *Firmicutes/Bacteroidetes* ratio after nine weeks of high-intensity interval training in healthy males.

In conclusion, this study further supports the significance of *Firmicutes* and *Bacteroidetes* and their ratio as a scaffold of a microbiologically healthy gut and, consequently, of the body's wellbeing, similarly to a conventional human structural organ. Reference intervals at every taxonomic level can be used as a reference for verifying whether a single subject or sample belongs to the microbiological community defined in this study. Reference intervals here reported refer to a sample of subjects who do not self-report clear disease symptoms; however, it will be interesting to use these reference intervals for future studies to verify whether samples from patients with specific diseases may or may not have a different microbial community. In this sense, it will then be possible to define the sensitivity and specificity of the reported reference intervals according to specific pathologies. This can be done either at the univariate level (*e.g.,* genus by genus) to verify whether each specific abundance is included within the reference interval or using a multivariate approach. In the latter case, the non-parametric approach of the Mahalanobis distance can be used. In this approach, the ranks of the abundances are used rather than the single absolute values^42^.

The association between the richness and diversity of gut microbiota and health has been demonstrated by Rinninella et al.^1^, although it appears difficult to identify a unique optimal gut microbiota composition. The main contribution of the present study is to help identify the existence, within the healthy Italian population, of a commonly distributed, constantly present, principal microbiological pattern, thus suggesting the presence of a sort of microbiological framework or scaffolding. In our view, this finding reinforces the concept that the human intestinal microbiota, with its morpho-functional and pathophysiological aspects, represents a real organ. Indeed, like anatomical organs, the intestinal microbiota may have individual variability; however, such variability must not substantially alter the fundamental framework of a healthy microbiota to ensure its correct functioning. Possible differences in the gut microbiota composition, diversity, and richness among individuals with the same ethnicity, residing in different Italian regions, or with different lifestyles only marginally affect the composition of the two main microbiological clusters identified in the present study. Taken together, our data highlight the significance of studies on population-specific variations in human microbiota composition. Nevertheless, at the same time, the present investigation underscores the need for variability studies to be able to consider even minimal variations in the intestinal microbiological population, given that, at least in the healthy population, there is a significant and reproducible presence of well-defined groups of bacteria, which represent a constant scaffold, or framework. In the next future, it will be crucial to share these data coming from different research groups in order to implement the “normal” range values or to build an algorithm capable of translating the composition of the microbiota associated with diseases states and of suggesting any dietary, pharmacological or lifestyle interventions in order to recover the state of eubiosis. Moreover, integrating these data with metabolomics and genetic variants could improve patient management. Again, this approach to studying the intestinal microbiota calls to mind studies focusing on human structural organs. Indeed, in our view, such an approach to the microbiota could help scientists to better design experimental plans and set up strategies based on precision tailored microbiota engineering.

## Methods

### Participants

A total of 148 control subjects from 17 Italian regions were recruited by the medical board, some of whose members were contributing authors of this work. The subjects, 69 males and 79 females, ranging in age from 23 to 57, were recruited from different Italian universities under the supervision of the University of Urbino Carlo Bo (Ethics Committee approval no. 34_2021). All the participating institutions followed the same pre-analytical and analytical procedures. All the subjects agreed to participate according to the ethical guidelines of the 2013 Declaration of Helsinki and signed written informed consent. The volunteer participants were selected to create a model of the Italian adult Caucasian population adequately represented in terms of gender, age, geographical origin, and place of residence (city or countryside) and that falls within the criteria of WHO definition of a "healthy" state of “complete physical, mental, and social well-being, not merely the absence of disease or infirmity”. In detail, the medical board evaluated each subject's complete medical history in order to exclude those who did not meet the study's inclusion criteria. The following subjects were excluded: those being treated with antibiotics or other drugs, those consuming probiotics, and those having a known history of inflammatory bowel disease, systemic disease, other autoimmune, metabolic, or psychiatric disorders or cancer. A questionnaire was then administered to each participant to collect the following information: body mass index, dietary habits, contact with farm animals or pets, smoking and physical activity habits, alcohol consumption, breastfeeding). Dietary patterns were classified into the Mediterranean, vegetarian/vegan, and others; the routine use of probiotics was also assessed. Furthermore, lactose and other food restrictions were evaluated, *i.e.,* voluntary limited consumption or exclusion of specific food groups (mainly dried or fresh vegetables and derivatives).

### Sample collection and DNA extraction

Samples were collected over two years, from fall 2017 to spring 2019. Fresh stool samples were collected within tubes containing a DNA stabilization buffer (Canvax Biotech) from each participant. In order to reduce any possible bias, pre-analytical and analytical procedures were performed at only one centre, according to our previously published study^9^. QIAamp DNA Stool Mini Kit (Qiagen, Milan, Italy) was utilized to perform total DNA extraction starting from 250 µL of each sample following the manufacturer's protocol. Once collected in the stabilizing liquid, samples were processed according to standardized times, usually not exceeding 5 days from the withdrawal; when was not possible to process the samples immediately at the arrival in the laboratory, they were stored at − 80° until processing, after assessing DNA concentration and purity.

### 16S rRNA gene sequence data processing

The Illumina 16S Metagenomic Sequencing Library Preparation for high-throughput sequencing was performed as follows: 12.5 ng of each DNA extract was employed for the amplification of the V3–V4 hypervariable regions of the bacterial 16S ribosomal RNA (rRNA) gene, using the following primers with Illumina adapters (underlined):

Forward primer (341F):

5′-TCGTCGGCAGCGTCAGATGTGTATAAGAGACAGCCTACGGGNGGCWGCAG

Reverse primer (785R):

5′-GTCTCGTGGGCTCGGAGATGTGTATAAGAGACAGGACTACHVGGGTATCTAATCC

As reported in Klindworth et al.^43^. Agencourt AMPure XP beads (Beckman Coulter, Milan, Italy) were used to purify PCR amplicons. The amplicons were then used for a second PCR in order to barcode the libraries using the Illumina dual-index system (Nextera XT Index Kit, Illumina Inc., San Diego, CA, USA) necessary for multiplexing. Following a second purification step, the eluted DNA products were quantified using the Qubit dsDNA BR Kit assay, diluted to 4 nM and pooled. The purified DNA products were then subjected to an additional PCR to attach dual Illumina indices (Nextera XT Index Kit, Illumina Inc., San Diego, CA, USA) necessary for multiplexing. Paired-end sequencing (2 × 300 cycles) was carried out using an Illumina MiSeq instrument (Illumina Inc.) according to the manufacturer's instructions. Sequences were demultiplexed based on index sequences, and FASTQ files were generated. FASTQ raw sequencing data were imported into QIIME2 v.2021.2^44^ environments, and then Illumina primers were removed using q2-cutadapt plugin in trim-paired mode^45^. Trimmed sequences were denoised in paired-end mode using q2-dada2 plugin^46^. The assignment of taxonomy to amplicon sequence variants (ASVs) was performed with q2-feature-classifier plugin^47^ against the pre-trained Naïve Bayes classifier SILVA 138 99% operational taxonomic units (OTUs) full-length sequence dataset^49^.

### Statistical analyses

The pan-microbiota (total observed richness in all samples) was determined in subsets of increasing size composed of randomly chosen samples (250 repetitions for each sample size). A collector’s curve, i.e., the total number of observed genera with increasing numbers of samples collected, was subsequently calculated (chronological order) (10 repetitions for each sample size), according to Falony et al.^50^. Once the sample's representativeness was checked, and it was noted whether the abundances showed very dispersed values, the presence of any homogeneous subgroups within the sample was verified. The *vegdist* function (*vegan* R package) was used to calculate Bray–Curtis distance, and the *kmeans* function was used to create the clusters^51^. The elbow and silhouette methods were used for determining the optimal clusters. Permutational multivariate analysis of variance (PERMANOVA) was performed on the Bray–Curtis distance matrix to determine if the gut microbiota structure differed between the two clusters, considering phyla, families, and genera. The *adonis2* function of the *vegan* R package was used. A heatmap was built as a graphical representation of the most abundant (representing 99% of total abundance) phyla using the *pheatmap* R package^52^. A chi-square test, or Fisher exact test when at least one class had n < 5, was used to test differences in microbiota composition and participant characteristics between the two clusters. The results are presented with *p*(χ^2^) and Cramer’s V. V values should be interpreted as > 0.5 = high association, 0.3 to 0.5 = moderate association, 0.1 to 0.3 = low association, 0 to 0.1 = little or no association. Richness (OTUs number) and Shannon's effective number were calculated using the *vegan* R package. A non-parametric method was used to calculate the reference intervals related to phyla, families, and genera. The 90% confidence intervals relative to the 95% lower and upper limits of the reference intervals were calculated using the bootstrap method according to the NCCLS Guidance Document C28A2^53^. The *referenceIntervals* R package was used^54^. The significance of differences in the abundance of phyla, families, and genera between clusters was tested using the Mann–Whitney test: *P* values from all statistical tests were adjusted for multiple comparisons within each taxonomic level, controlling the False Discovery Rate (FDR) (*FSA* R package) at level 0.05 using the Benjamini–Hochberg step-up procedure^55^. A graphical display of a non-parametric correlation matrix, based on Spearman's R, ordered according to hierarchical clustering, was obtained using the *corrplot* R package. All the analyses were conducted using Microsoft Excel 16, Prism 8 (GraphPad Software, San Diego, CA), and R Studio 3.6.2.

### Ethics approval

The study was approved by the Urbino University Ethics Committee (approval number 34_2021).

## Supplementary Information

Supplementary Figures.

## Data Availability

Additional data is available in the supplementary material, and the datasets generated during the current study will be available upon request to the corresponding author.

## References

[1] Rinninella E (2019). Food components and dietary habits: Keys for a healthy gut microbiota composition. Nutrients.

[2] Illiano P, Brambilla R, Parolini C (2020). The mutual interplay of gut microbiota, diet and human disease. FEBS J..

[3] Ventura M (2009). Microbial diversity in the human intestine and novel insights from metagenomics. Front. Biosci. (Landmark Ed.).

[4] Grenham S, Clarke G, Cryan JF, Dinan TG (2011). Brain-gut-microbe communication in health and disease. Front. Physiol..

[5] Yadav M, Verma MK, Chauhan NS (2018). A review of metabolic potential of human gut microbiome in human nutrition. Arch. Microbiol..

[6] Rahayu ES (2019). Gut microbiota profile in healthy Indonesians. World J. Gastroenterol..

[7] Donati Zeppa S (2019). Mutual interactions among exercise, sport supplements and microbiota. Nutrients.

[8] Klement RJ, Pazienza V (2019). Impact of different types of diet on gut microbiota profiles and cancer prevention and treatment. Medicina (Kaunas).

[9] Fontana A (2019). Gut microbiota profiles differ among individuals depending on their region of origin: An Italian pilot study. Int. J. Environ. Res. Public Health.

[10] He Y (2018). Author Correction: Regional variation limits applications of healthy gut microbiome reference ranges and disease models. Nat. Med..

[11] Senghor B, Sokhna C, Ruimy R, Lagier JC (2018). Gut microbiota diversity according to dietary habits and geographical provenance. Hum. Microbiome J..

[12] Panebianco C, Potenza A, Andriulli A, Pazienza V (2018). Exploring the microbiota to better understand gastrointestinal cancers physiology. Clin. Chem. Lab. Med..

[13] Picchianti-Diamanti A (2018). Analysis of gut microbiota in rheumatoid arthritis patients: Disease-related dysbiosis and modifications induced by etanercept. Int. J. Mol. Sci..

[14] Wang B, Yao M, Lv L, Ling Z, Li L (2017). The human microbiota in health and disease. Engineering.

[15] Parfrey LW, Knight R (2012). Spatial and temporal variability of the human microbiota. Clin. Microbiol. Infect..

[16] Rinninella E (2019). What is the healthy gut microbiota composition? A changing ecosystem across age, environment, diet, and diseases. Microorganisms.

[17] De Luca F, Shoenfeld Y (2019). The microbiome in autoimmune diseases. Clin. Exp. Immunol..

[18] Nagao-Kitamoto H, Kitamoto S, Kuffa P, Kamada N (2016). Pathogenic role of the gut microbiota in gastrointestinal diseases. Intest. Res..

[19] Flowers SA, Ward KM, Clark CT (2020). The gut microbiome in bipolar disorder and pharmacotherapy management. Neuropsychobiology.

[20] Huang TT (2019). Current understanding of gut microbiota in mood disorders: An update of human studies. Front. Genet..

[21] Tang WHW, Backhed F, Landmesser U, Hazen SL (2019). Intestinal microbiota in cardiovascular health and disease: JACC state-of-the-art review. J. Am. Coll. Cardiol..

[22] Eckburg PB (2005). Diversity of the human intestinal microbial flora. Science.

[23] Arumugam M (2011). Enterotypes of the human gut microbiome. Nature.

[24] Gallardo-Becerra L (2020). Metatranscriptomic analysis to define the Secrebiome, and 16S rRNA profiling of the gut microbiome in obesity and metabolic syndrome of Mexican children. Microb. Cell Fact..

[25] Gerard P (2016). Gut microbiota and obesity. Cell. Mol. Life Sci..

[26] Cooper P (2013). Patent human infections with the whipworm, *Trichuris trichiura*, are not associated with alterations in the faecal microbiota. PLoS ONE.

[27] Koeth RA (2013). Intestinal microbiota metabolism of l-carnitine, a nutrient in red meat, promotes atherosclerosis. Nat. Med..

[28] Aya V, Florez A, Perez L, Ramirez JD (2021). Association between physical activity and changes in intestinal microbiota composition: A systematic review. PLoS ONE.

[29] Nishijima S (2016). The gut microbiome of healthy Japanese and its microbial and functional uniqueness. DNA Res..

[30] De Filippo C (2010). Impact of diet in shaping gut microbiota revealed by a comparative study in children from Europe and rural Africa. Proc. Natl. Acad. Sci. U. S. A..

[31] de Alencar H (2020). The relationship between the commensal microbiota levels and Crohn's disease activity. JGH Open.

[32] Mazzarella G (2017). Pathogenic role of associated adherent-invasive *Escherichia coli* in Crohn's disease. J. Cell. Physiol..

[33] Pittayanon R (2020). Differences in gut microbiota in patients with vs without inflammatory bowel diseases: A systematic review. Gastroenterology.

[34] Perna A (2020). Adherent-invasive *Escherichia coli* (AIEC): Cause or consequence of inflammation, dysbiosis, and rupture of cellular joints in patients with IBD?. J. Cell. Physiol..

[35] Merra G (2020). Influence of Mediterranean diet on human gut microbiota. Nutrients.

[36] McDonald D (2018). American gut: An open platform for citizen science microbiome research. mSystems.

[37] Johnson AJ (2019). Daily sampling reveals personalized diet-microbiome associations in humans. Cell Host Microbe.

[38] Han M (2020). Stratification of athletes' gut microbiota: The multifaceted hubs associated with dietary factors, physical characteristics and performance. Gut Microbes.

[39] Clarke SF (2014). Exercise and associated dietary extremes impact on gut microbial diversity. Gut.

[40] Huang J (2020). Six-week exercise training with dietary restriction improves central hemodynamics associated with altered gut microbiota in adolescents with obesity. Front. Endocrinol. (Lausanne).

[41] Donati Zeppa S (2021). Nine weeks of high-intensity indoor cycling training induced changes in the microbiota composition in non-athlete healthy male college students. J. Int. Soc. Sports Nutr..

[42] Nanna MJ (2002). Hotelling's T2 vs. the rank transform with real Likert data. J. Mod. Appl. Stat. Methods.

[43] Klindworth A (2013). Evaluation of general 16S ribosomal RNA gene PCR primers for classical and next-generation sequencing-based diversity studies. Nucl. Acids Res..

[44] Bolyen E (2019). Reproducible, interactive, scalable and extensible microbiome data science using QIIME 2. Nat. Biotechnol..

[45] Martin M (2011). Cutadapt removes adapter sequences from high-throughput sequencing reads. EMBnet.journal.

[46] Callahan BJ (2016). DADA2: High-resolution sample inference from Illumina amplicon data. Nat. Methods.

[47] Bokulich NA (2018). Optimizing taxonomic classification of marker-gene amplicon sequences with QIIME 2's q2-feature-classifier plugin. Microbiome.

[48] Bokulich NA (2021). Zenodo.

[49] Quast C (2013). The SILVA ribosomal RNA gene database project: Improved data processing and web-based tools. Nucl. Acids Res..

[50] Falony G (2016). Population-level analysis of gut microbiome variation. Science.

[51] *vegan: Community Ecology Package* (2008).

[52] *pheatmap: Pretty Heatmaps* (2015).

[53] NCCLS. *How to Define and Determine Reference Intervals in the Clinical Laboratory; Approved Guideline—Second Edition. NCCLS Document C28-A2*, vol. 20 (2000).

[54] *referenceIntervals: Reference Intervals* (2020).

[55] *FSA: Fisheries Stock Analysis* (2021).

