# Supplementary material for: A proposal for the reference intervals of the Italian microbiota “scaffold” in healthy adults
Source: Sci Rep. 2022 Mar 10;12:3952. doi: 10.1038/s41598-022-08000-x (PMC8913673; doi:10.1038/s41598-022-08000-x)
Supplement: Supplementary file 1 — Supplementary Figures. [file 41598_2022_8000_MOESM1_ESM.docx]

**Supplementary Figure 1.** The total number of genera comprising the human gut microbiota, which increases with the number of individuals.

**
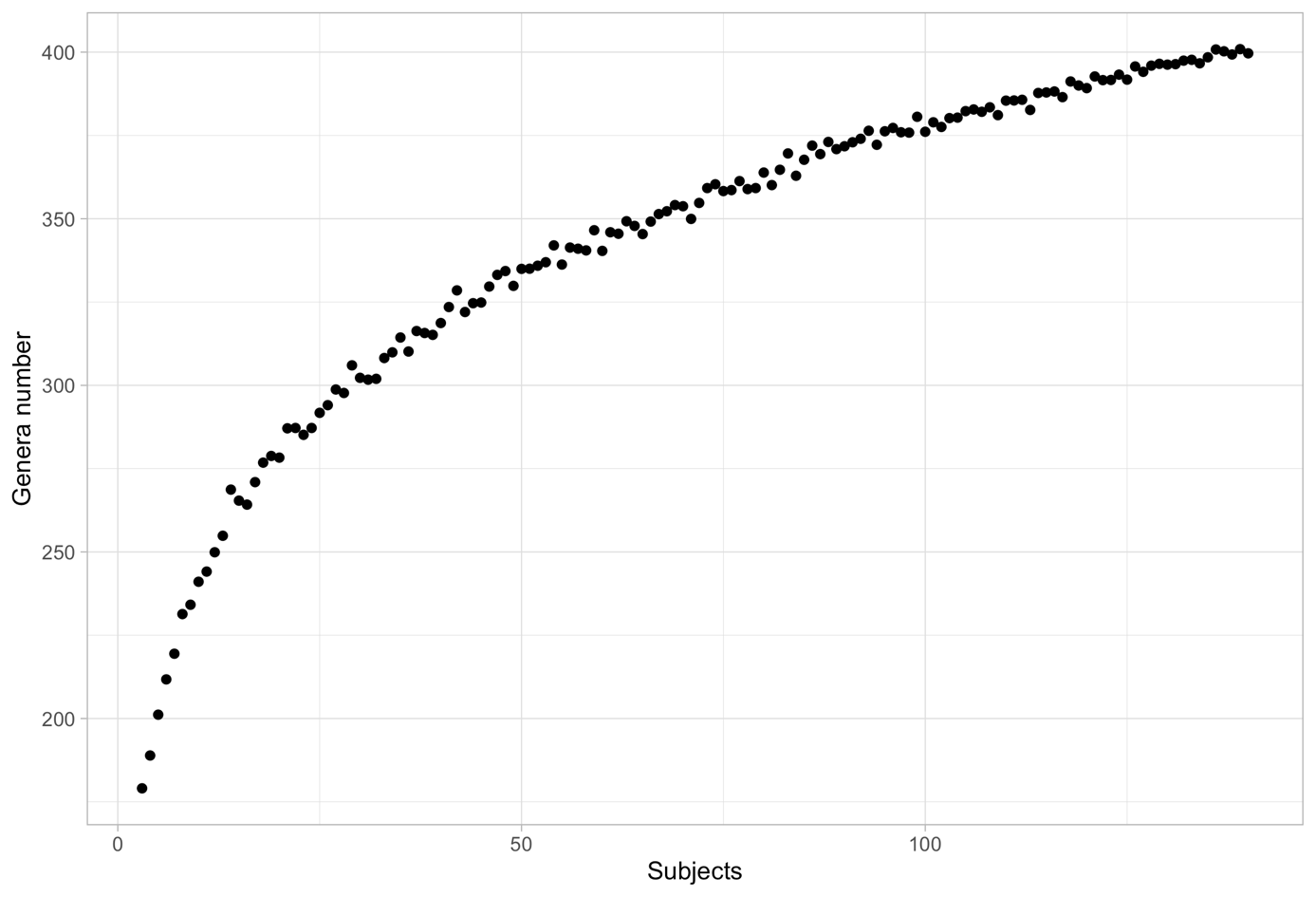
**

**Supplementary Figure 2.** Clustering validation using the Elbow (left) and Silhouette (right) methods. Both methods found two clusters to be the best solution.
